# Preoperative Immune-Inflammatory Status of the Patients With Newly-Diagnosed Glioblastoma – Could It Genuinely Predict Their Survival?

**DOI:** 10.7759/cureus.43802

**Published:** 2023-08-20

**Authors:** Georgiana M Serban, Corina I Tamas, Flaviu Tamas, Adrian F Balasa

**Affiliations:** 1 Anesthesiology and Critical Care Clinic, Emergency County Hospital, Targu Mures, ROU; 2 Neurosurgery Clinic, Emergency County Hospital, Targu Mures, ROU; 3 Neurosurgery, George Emil Palade University of Medicine, Pharmacy, Science, and Technology, Targu Mures, ROU; 4 Neurosurgery, Emergency County Hospital, Targu Mures, ROU

**Keywords:** systemic immune response index, systemic immune-inflammatory index, monocyte-to-lymphocyte ratio, platelet-to-lymphocyte ratio, neutrophils-to-lymphocyte ratio, overall survival, prognostic markers, glioblastoma multiforme

## Abstract

Background: Glioblastoma multiforme (GBM) is the most aggressive brain tumor affecting adult patients, with an extremely reduced overall survival despite rapid diagnosis and treatment. Therefore, it is crucial to establish accurate and affordable markers that allow an individualized approach to GBM patients. Serum biomarkers could be the most accessible, as complete blood counts should be performed on all GBM patients before undergoing any surgical and/or pharmacological treatment. However, their prognostic role is still unclear. Our study aims to assess the influence of various hematological markers of inflammation in predicting the outcome of GBM patients.

Material and methods: We retrospectively analyzed all adult patients diagnosed with primary glioblastoma in the Neurosurgery Department of the Emergency Clinical County Hospital of Târgu Mureș, Romania, from January 2017 until December 2019. We aimed to discover whether the immune/inflammatory status of the patients before receiving any kind of pharmacological or surgical treatment influenced their overall survival.

Results: Our study showed that pre-therapeutic elevated white blood count could predict reduced overall survival in not otherwise specified subtype (NOS) of GBMs (HR 0.4153, 95% CI 0.1825-0.9449, p 0.0362). Furthermore, patients with increased systemic immune response index (SIRI) had much larger tumors at the time of diagnosis (p 0.0359). In wild type, isocitrate dehydrogenase subpopulation (IDHwt), the higher values of neutrophil-to-lymphocyte ratio (NLR, p 0.0412), platelet-to-lymphocyte ratio (PLR, p 0.0376) and monocyte-to-lymphocyte ratio (MLR, p 0.0412) were related to more advanced age at the moment of diagnosis. Moreover, our results revealed a weakly positive association between tumor size and NLR values in the NOS group (Spearman r 0.3212, p 0.0493).

Conclusions: Our study does not provide enough evidence for the immune/inflammatory status of GBM patients to be used as an efficient prognostic marker to guide the therapeutic approach.

## Introduction

Glioblastoma multiforme (GBM), a WHO grade IV glioma, is the most frequent malignant cerebral tumor occurring in adult patients and has a notoriously limited survival of up to 21 months despite the standard of care being applied [1]. The currently worldwide accepted treatment, also known as the Stupp protocol, consists of extensive surgical resection, adjuvant radio- and chemotherapy. However, the overall survival greatly differs among patients [2]. This is why it is paramount to establish reliable prognostic markers to guide physicians toward a more personalized approach. Numerous factors either related to patients (e.g., age at diagnosis, preoperative clinical performance quantified by Karnofsky Performance Scale), to treatment (e.g., the extent of surgical removal, response to adjuvant therapies, a period without recurrences), or to the tumor itself (e.g., localization, volume, imagistic heterogeneity, molecular features - IDH1 status, MGMT gene promoter methylation status, histopathological characteristics - Ki67 index) have been considered, yet they have suboptimal accuracy [2,3].

Peripheral blood biomarkers that have demonstrated their role in grading and predicting the outcome of various types of cancer, namely lung, renal and gastric malignancies, have lately emerged as affordable and readily accessible alternatives [4]. Inflammation is deeply involved in creating a favorable environment for tumors to thrive by promoting cell proliferation and survival and increasing their vascular supply [5,6]. Due to the excessive production of granulocyte colony-stimulating factor (G-CSF) stimulated by tumor cells in glioma patients, peripheral blood assays show increased neutrophilia associated with lymphopenia [6]. The balance between these two, quantified by the neutrophil-to-lymphocyte ratio (NLR), might reflect the host reaction to cancer development: neutrophilic predominance over lymphocytes suggests an overwhelming inflammatory response, possibly at the expense of an ineffective immunological state [5]. Other hematological biomarkers have been utilized as well, like monocytes and eosinophils. For instance, lymphocytes and eosinophils share an anti-carcinogenic response and, therefore, a better prognosis [4,7,8]. Consequently, hematological indicators of systemic inflammation based on these cell counts have been formulated, such as neutrophils-to-lymphocyte ratio (NLR), platelet-to-lymphocyte ratio (PLR), monocyte-to-lymphocyte ratio (MLR), systemic immune-inflammatory index (SII) and systemic immune response index (SIRI) [2,3,9,10]. Unfortunately, their prognostic reliability in clinical practice is questionable, as prior studies have shown conflicting results.

On these grounds, our retrospective study aimed to evaluate the role of the abovementioned hematological indicators of systemic inflammation, as well as the absolute number of various cells (platelets, white blood cells, lymphocytes, neutrophils) in predicting the evolution of the disease in patients with newly-diagnosed primary GBM before receiving any treatment.

## Materials and methods

Our retrospective study involved 89 adult patients hospitalized and diagnosed with primary GBM in the Neurosurgery Department of the Emergency Clinical County Hospital of Târgu Mureș, Romania, from January 2017 until December 2019. This study was approved by the Local Ethics Committee of the Emergency Clinical County Hospital of Târgu Mureș, Romania (no. 7540/05.04.2023). The inclusion criteria consist of adult patients with confirmed histopathological diagnoses of primary GB. The patients with secondary GBM, previous surgical, adjuvant, or steroid treatment, with prior records of malignancies or auto-immune disorders, or whose clinical and imagistic data could not be found were excluded from the study. All patients received standard treatment according to the Stupp protocol.

Using the patient's medical records stored in the Hospital Information System, the following data were acquired: gender and age of the patients at the time of diagnosis, size of the tumor (defined as the largest measurement in the axial plane based on the MRI performed at the time of diagnosis and expressed in millimeters), localization of the tumor, complete blood count (absolute numbers of white blood cells, platelets, lymphocytes, neutrophils, monocytes, and eosinophils). According to written and verbal information provided by the patient's relatives, we also obtained data related to pre-treatment clinical status quantified by the Karnofsky Performance Scale (KPS), a molecular subtype of the tumor and overall survival (defined as the time in months from the time of diagnosis to the time of death).

The data were gathered and statistically analyzed using GraphPad and MedCalc software programs. We further presented the numerical data according to their distribution assessed by the Kolmogorov-Smirnov normality test as mean ± standard deviation for those with Gaussian distribution and as median ± interquartile range for those with non-parametrical distribution, respectively. When comparing two samples depending on the data type (paired or unpaired), we either applied the variants of t-student tests for normally distributed data or Wilcoxon and Mann-Whitney test for data with non-Gaussian distribution. We assessed potential correlations between two groups using Pearson's or Spearman's correlation coefficients in conformity with data distribution. Overall survival rates were calculated using Kaplan-Meier survival curves. We sought statistical differences between two survival curves using a log-rank test and assessed the influence of independent variables on overall survival utilizing the Cox regression test. We determined the cut-off values based on receiver operating characteristic (ROC) curve analysis. We established that a p-value lower than 0.05 was considered statistically significant with a confidence interval of 95%.

## Results

Eighty-nine patients with newly diagnosed GBMs were initially included in our study. After applying our exclusion criteria, 15 patients were eliminated from further statistical analysis: two patients with previous surgical treatment, six with prior steroid treatment, and seven with follow-up data could not be obtained. Among the remaining 74 cases, 35 were women (47.3%) and 39 men (52.7%), with a median age of 61.5 years. The mean tumor dimension was 45.39±15.6 mm. Regarding tumor localization, there was a slight predominance of the left side compared to the right side of the brain (39 vs. 35). Most frequently, GBMs developed in temporal and frontal regions (28 cases each), while the occipital area was the least affected (only one patient out of 74).

Regarding the clinical status of the patients when first admitted to the hospital, the median preoperative KPS was 80. The survival range broadly varied between two weeks to more than 62 months, with a mean overall survival of ten months. At the moment of data gathering (May 2023), only three patients were still alive: two cases of not otherwise specified subtypes of GBMs (NOS) and one of epitheloid GBM. Regarding histopathological classification, the most encountered molecular subtype was NOS GBMs (38 cases), followed by epitheloid subtypes (19 cases). The least common subpopulation belonged to the mutant isocitrate dehydrogenase (IDHm) subtype with only three patients. Eleven patients were diagnosed with wild-type isocitrate dehydrogenase (IDHwt) GBMs.

Utilizing complete blood count absolute values, we further calculated various ratios as serum biomarkers describing the immune-inflammatory status of each patient before receiving any surgical or pharmacological treatment, such as neutrophil-to-lymphocyte ratio (NLR) (absolute count of neutrophils/ absolute count of lymphocytes), platelet-to-lymphocyte ratio (PLR) (absolute count of platelets / absolute count of lymphocytes), eosinophil-to-lymphocyte ratio (ELR) (absolute count of eosinophils/ absolute count of lymphocytes), monocyte-to-lymphocyte ratio (MLR) (absolute count of monocytes/ absolute count of lymphocytes), systemic immune response index (SIRI) (absolute count of neutrophils × absolute count of monocytes/ absolute count of lymphocytes) and systemic immune-inflammatory index (SII) (absolute count of neutrophils × absolute count of platelets/ absolute count of lymphocytes). Using ROC curve analysis on our GBM cohort, we defined a cut-off value for each serum biomarker, including the total number of white blood cells (WBC), lymphocytes (LYMPH), neutrophils (NEUTR) and platelets (PLT): WBC 9.53, LYMPH 1.3, NEUTR 8.3, PLT 235, NLR 3.14, PLR 150, MLR 0.39, ELR 0.0226, SII 684, SIRI 2.79.We used the abovementioned cut-off values for statistical analysis when comparing means or medians in the entire GBM cohort or among different molecular subpopulations.

Furthermore, we compared the means/medians (in dependence on data distribution) of patients' age, preoperative KPS, survival, and tumor size according to the cut-off value for each serum biomarker. When assessing all GBM cases, irrespective of the molecular subtypes, we found a statistically significant difference in tumor size related to SIRI: patients with increased SIRI values had much larger tumors at the time of diagnosis (p 0.0359). Moreover, patients with higher SIRI tended to have a more severe onset of the disease, as suggested by preoperative KPS, yet this trend was not supported by statistical analysis (p 0.055). No other significant differences have been noted (Table 1).

**Table 1:** All GBM population - statistical comparisons between means/medians, with p<0.05 considered statistically significant (data with Gaussian distribution are presented as arithmetic means ± standard deviation and data with non-Gaussian distribution are presented as medians (Q1; Q3)). GBM - glioblastoma multiforme; NLR - neutrophil-to-lymphocyte ratio; PLR - platelet-to-lymphocyte ratio; MLR - monocyte-to-lymphocyte ratio; ELR - eosinophil-to-lymphocyte ratio; SII - systemic immune-inflammatory index; SIRI - systemic immune response index; WBC - absolute count of white blood cells; LYMPH - absolute count of lymphocytes; PLT - absolute count of platelets; NEUTR - absolute count of neutrophils

All GBMs		Age (years)	p value	Dimension (mm)	p value	KPS	p value	Survival (months)	p value
NLR	<3.14	56.26±13.39	0.3189	41.105±16.36	0.1879	80 (60;87.5	0.6318	3 (2;16)	0.995
	≥3.14	61 (50;69)		46.875±15.2		80 (70;90)		5 (3;7.75)	
PLR	<150	57.83±12.66	0.4751	45.3±15.13	0.8160	80 (65;90)	0.9244	4.5 (2;12)	0.4734
	≥150	63 (50;69)		43.9 (32;54)		80 (70;90)		4 (2;7)	
MLR	<0.39	55.51±13.42	0.1229	41.81±15.58	0.1382	80 (80;90)	0.2632	6 (2.25;10.75)	0.3759
	≥0.39	60.4±11.89		47.45±15.4		80 (60;90)		4 (2;7.75)	
ELR	<0.0226	60 (46;69)	0.7008	45.75±15.84	0.6308	80 (70;90)	0.9377	5 (3;10)	0.5241
	≥0.0226	60.31±8.86		43.86±15.22		80 (60;90)		3 (2;11)	
SIRI	<2.79	61.5 (46;67)	0.1965	40.35±14.34	0.0359	80 (80;90)	0.4131	6 (3;16)	0.1362
	≥2.79	60.06±11.95		48.12±15.71		80 (65;90)		4 (2;7)	
SII	<684	57.33±12.14	0.5097	43.11±17.02	0.5603	80 (60;87.5)	0.7919	5 (2;19.25)	0.3584
	≥684	62 (50;69)		45.97±15.32		80 (70;90)		4 (2.25;7.75)	
WBC	<9.53	58.13±12.82	0.8327	42.47±16.39	0.3157	80 (70;90)	0.6602	6.5 (3;13)	0.1329
	≥9.53	58.82±12.63		46.62±15.25		80 (60;90)		4 (2;8)	
LYMPH	<1.3	62 (51.25;69.75)	0.4042	47.98±17.27	0.1828	80 (62.5;90)	0.3741	5 (2.25;7)	0.6478
	≥1.3	57.66±12.74		43.06±13.75		80 (70;80)		4 (2;12.5)	
PLT	<235	63 (47;69)	0.8094	47.18±15.87	0.2449	80 (62.5;90)	0.9594	5 (2;12.5)	0.2219
	≥235	58.54±11.48		42.91±15.12		80 (70;90)		3 (2.25;8.5)	
NEUTR	<8.3	63 (51.25;67)	0.999	40.9±15.4	0.0622	80 (72.5;87.5)	0.6764	6 (2.25;12.25)	0.183
	≥8.3	58.48±12.96		47.97±15.28		80 (60;90)		4 (2;7)	

We applied the same analysis on each molecular subpopulation (epitheloid - Table 2, IDHwt - Table 3 and NOS - Table 4).

**Table 2:** Epitheloid GBM subpopulation - statistical comparisons between means/medians, with p<0.05 considered statistically significant (data with Gaussian distribution are presented as arithmetic means ± standard deviation and data with non-Gaussian distribution are presented as medians (Q1; Q3)). GBM - glioblastoma multiforme; NLR - neutrophil-to-lymphocyte ratio; PLR - platelet-to-lymphocyte ratio; MLR - monocyte-to-lymphocyte ratio; ELR - eosinophil-to-lymphocyte ratio; SII - systemic immune-inflammatory index; SIRI - systemic immune response index; WBC - absolute count of white blood cells; LYMPH - absolute count of lymphocytes; PLT - absolute count of platelets; NEUTR - absolute count of neutrophils

Epitheloid GBMs		Age (years)	p value	Dimension (mm)	p value	KPS	p value	Survival (months)	p value
NLR	<3.14	56.28±15.15	0.7321	40.71±17.53	0.7148	71.42±14.63	0.4983	11.57±15.79	0.6406
	≥3.14	58.58±10.84		43.4±8.73		80 (65;80)		6 (4;9)	
PLR	<150	60.81±9.31	0.2485	43.9±15.66	0.4971	72.72±14.2	0.3319	5 (2.25;13.75)	0.8035
	≥150	53.5±15.01		40.35±5.4		80 (70;85)		6 (3.5;10.5)	
MLR	<0.39	55.5±13.64	0.4103	38.88±14.86	0.1847	80 (80;90)	0.0975	5.5 (3;10)	0.8057
	≥0.39	60.2±10.67		46.33±7.61		70 (60;80)		9.88±11.29	
ELR	<0.0226	56±14.17	0.3674	42.15±9.37	0.922	80 (65;80)	0.588	6 (4;10.5)	0.3728
	≥0.0226	60.7±8.01		42.85±17.06		72.85±17.04		3 (2;14)	
SIRI	<2.79	54.3±13.54	0.1993	38.08±10.42	0.1131	80 (80;90)	0.055	10.8±13.27	0.9673
	≥2.79	61.55±9.96		47.22±12.94		68.88±11.66		6 (3.75;8)	
SII	<684	60.5±8.75	0.4505	38.33±13.32	0.3728	76.66±15.05	0.7787	3.5 (2;17)	0.5971
	≥684	56.46±13.67		44.29±11.84		80 (60;80)		6 (3.75;10.25)	
WBC	<9.53	55.87±13.87	0.6	39.12±12.66	0.8362	77.5±13.88	0.5083	11.25±14.57	0.9339
	≥9.53	59.09±11.39		43.8 (38;45.75)		80 (60;80)		6 (3.25;9.75)	
LYMPH	<1.3	59.42±9.84	0.6292	44.42±9.14	0.5566	74.28±15.11	0.7865	5.28±3.03	0.1181
	≥1.3	56.75±13.75		41.23±14.04		80 (65;80)		12.5±14.39	
PLT	<235	59.83±10.54	0.3943	40.58±12.56	0.6116	80 (65;80)	0.8567	6 (3;13.5)	0.5243
	≥235	54.14±14.87		45 (39.45;45.75		72.85±16.03		4 (3;9)	
NEUTR	<8.3	55.87±13.87	0.6	39.12±12.66	0.8362	77.5±13.88	0.5083	11.25±14.57	0.9339
	≥8.3	59.09±11.39		43.8 (38;45.75)		80 (60;80)		6 (3.25;9.75)	

**Table 3:** IDHwt GBM subpopulation - statistical comparisons between means/medians, with p<0.05 considered statistically significant (data with Gaussian distribution are presented as arithmetic means ± standard deviation and data with non-Gaussian distribution are presented as medians (Q1; Q3)). IDHwt - isocitrate dehydrogenase  wild type; GBM - glioblastoma multiforme; NLR - neutrophil-to-lymphocyte ratio; PLR - platelet-to-lymphocyte ratio; MLR - monocyte-to-lymphocyte ratio; ELR - eosinophil-to-lymphocyte ratio; SII - systemic immune-inflammatory index; SIRI - systemic immune response index; WBC - absolute count of white blood cells; LYMPH - absolute count of lymphocytes; PLT - absolute count of platelets; NEUTR - absolute count of neutrophils

IDHwt GBMs		Age (years)	p value	Dimension (mm)	p value	KPS	p value	Survival (months)	p value
NLR	<3.14	44 (35.75;46.25)	0.0412	42 (36.75;54)	0.5384	90 (82.5;97.5)	0.1978	3 (1.5;15.75)	0.6824
	≥3.14	67±11.32		40.25±16.51		81.25±8.34		7.31±4.72	
PLR	<150	45.5 (38.5;55.5)	0.0376	38.5 (32.5;50)	0.9244	90 (85;95)	0.0912	5.5 (2;14)	1.00
	≥150	70 (67.5;74.5)		41.71±17.26		80±8.16		7.21±5.09	
MLR	<0.39	44 (35.75;46.25)	0.0412	42 (36.75;54)	0.5384	90 (82.5;97.5)	0.1978	3 (1.5;15.75)	0.6824
	≥0.39	67±11.32		40.25±16.51		81.25±8.34		7.31±4.72	
ELR	<0.0226	59.25±15.82	0.7758	42.25±16.025	0.7758	83.75±9.16	0.7758	7.43±7.29	0.4970
	≥0.0226	70 (48.25;73.75)		38.5 (28.75;49)		80 (75;90)		7 (7;8.5)	
SIRI	<2.79	56±16.59	0.4732	36.6±13.77	0.3365	84±11.4	0.9159	9.2±6.45	0.4355
	≥2.79	63.33±15.46		45.66±15.83		83.33±8.16		6.08±6.03	
SII	<684	58.33±16.02	0.722	46.33±16.41	0.2526	83.33±10.32	0.7734	6.91±6.9	0.7445
	≥684	62±16.76		35.8±12.11		90 (77.5;90)		8.2±5.76	
WBC	<9.53	66.5 (54;71)	1.00	36 (30;57)	1.00	80 (80;85)	0.6911	4.5 (1.75;7)	0.1849
	≥9.53	58.57±17.83		40.42±13.12		84.28±11.33		9.28±6.84	
LYMPH	<1.3	59.42±18.43	0.5708	44.14±17.8	0.5064	85.71±9.75	0.3205	8.78±7.4	0.4487
	≥1.3	65.5 (54;68)		36 (30;44)		80 (75;85)		5 (3.5;7)	
PLT	<235	66.16±10.43	0.1983	39.66±17.37	0.6633	81.66±7.52	0.4903	6.08±6.06	0.435
	≥235	52.6±18.68		43.8±13.04		86±11.4		9.2±6.41	
NEUTR	<8.3	58.8±19.058	0.8363	50.4±15.9	0.0955	80 (80;92.5)	0.6451	7.3±7.54	0.421
	≥8.3	61±13.986		34.16±10.06		81.66±9.832		8.9±7.06	

**Table 4:** NOS GBM subpopulation - statistical comparisons between means/medians, with p<0.05 considered statistically significant (data with Gaussian distribution are presented as arithmetic means ± standard deviation and data with non-Gaussian distribution are presented as medians (Q1; Q3)). NOS - not otherwise specified; GBM - glioblastoma multiforme; NLR - neutrophil-to-lymphocyte ratio; PLR - platelet-to-lymphocyte ratio; MLR - monocyte-to-lymphocyte ratio; ELR - eosinophil-to-lymphocyte ratio; SII - systemic immune-inflammatory index; SIRI - systemic immune response index; WBC - absolute count of white blood cells; LYMPH - absolute count of lymphocytes; PLT - absolute count of platelets; NEUTR - absolute count of neutrophils

NOS GBMs		Age (years)	p value	Dimension (mm)	p value	KPS	p value	Survival (months)	p value
NLR	<3.14	66 (58.75;67.5)	0.9863	40.11±18.17	0.2922	80 (57.5;82.5)	0.4183	11.11±13.08	0.8081
	≥3.14	59.55±12.56		47.52±15.81		80 (67.5;90)		3 (2;7.5)	
PLR	<150	60.29±13.1	0.8947	45.05±16.08	0.8113	80 (67.5;90)	0.6302	4 (2;13.5)	0.4232
	≥150	62 (53;69)		46.35±17.12		75.71±14.68		3 (2;5.5)	
MLR	<0.39	66 (48;68)	0.7651	43.5±18.81	0.6019	80 (70;85)	0.9487	5.5 (2;21)	0.6117
	≥0.39	60.5±11.45		46.81±15.53		80 (60;90)		3 (2;5)	
ELR	<0.0226	59.55±13.35	0.7033	47.08±17.06	0.4282	80 (62.5;90)	0.419	4 (2.25;9.25)	0.7947
	≥0.0226	60.9±7.98		42.54±15.12		79.09±18.14		3 (2;12)	
SIRI	<2.79	66 (49;67.5)	0.7579	40.41±17.78	0.1758	80 (57.5;90)	0.8966	13.71±13.84	0.2565
	≥2.79	60.4±11.67		48.55±15.35		80 (67.5;90)		3 (2;6.5)	
SII	<684	66 (50.25;68.25)	0.8357	45.24±21.52	0.9421	70±20	0.3543	7 (2.75;17.75)	0.3043
	≥684	59.93±12.23		45.89±15.53		80 (70;90)		3 (2;5)	
WBC	<9.53	58.2±12.88	0.617	44.74±18.85	0.837	77±19.46	0.8385	14.8±15.88	0.0238
	≥9.53	60.57±11.79		46.13±15.87		80 (60;90)		3 (2;5)	
LYMPH	<1.3	58.27±13.69	0.4295	49.36±18.21	0.2121	80 (60;90)	0.4721	3.5 (2;7)	0.8826
	≥1.3	61.45±10.27		42.53±14.39		80 (65;85)		3.5 (2;11.5)	
PLT	<235	66 (51.25;69)	0.8607	50.04±17.34	0.1108	80 (60;90)	0.9404	4 (2;19.75)	0.3452
	≥235	60.31±10.51		41.5±14.73		80 (70;90)		3 (2.25;6.5)	
NEUTR	<8.3	65 (50;67)	0.555	41.56±17.31	0.2194	80 (70;90)	0.8673	8 (3;22)	0.0691
	≥8.3	60.66±11.908		48.51±15.64		80 (60;90)		3 (2;4.5)	

We discovered a much lower overall survival in patients with increased WBC numbers in the NOS group (p 0.0238). Moreover, in the IDHwt subpopulation, the higher values of NLR (p 0.0412), PLR (p 0.0376), and MLR (p 0.0412) were related to more advanced age at the time of diagnosis.

We also assessed the statistical correlations between the serum biomarkers of the immune-inflammatory status and patients' age, preoperative KPS, and tumor size in patients having similar molecular GBM subtypes and in the total GBM population (see Table 5-8).

**Table 5:** Statistical correlations among different variables in all GBM patients. GBM - glioblastoma multiforme; NLR - neutrophil-to-lymphocyte ratio; PLR - platelet-to-lymphocyte ratio; MLR - monocyte-to-lymphocyte ratio; ELR - eosinophil-to-lymphocyte ratio; SII - systemic immune-inflammatory index; SIRI - systemic immune response index; WBC - absolute count of white blood cells; LYMPH - absolute count of lymphocytes; PLT - absolute count of platelets; NEUTR - absolute count of neutrophils

All GBMs	Age	Dimension	Preoperative KPSI
NLR	Spearman r 0.0497	Spearman r 0.1156	Spearman r 0.07168
	p 0.6731	p 0.3269	p 0.5439
	CI (-0.1807, 0.2753)	CI (-0.1161, 0.3353)	CI (-0.1662, 0.301)
MLR	Spearman r 0.07168,	Spearman r -0.2097,	Spearman r 0.07168,
	p 0.5439	p 0.0730	p 0.5439
	CI (-0.1662, 0.301)	CI (-0.4238, 0.02672)	CI (-0.1662, 0.301)
PLR	Spearman r 0.0497	Spearman r 0.1156	Spearman r 0.07168,
	p 0.6731	p 0.3269	p 0.5439
	CI (-0.1807, 0.2753)	CI (-0.1161, 0.3353)	CI (-0.1662, 0.301)
ELR	Spearman r 0.07168	Spearman r -0.2097	Spearman r 0.07168
	p 0.5439	p 0.0730	p 0.5439
	CI (-0.1662, 0.301)	CI (-0.4238, 0.02672)	CI (-0.1662, 0.301)
SII	Spearman r 0.07168	Spearman r -0.2097	Spearman r 0.07168
	p 0.5439	p 0.0730	p 0.5439
	CI (-0.1662, 0.301)	CI (-0.4238, 0.02672)	CI (-0.1662, 0.301)
SIRI	Spearman r 0.07168	Spearman r -0.2097	Spearman r 0.07168
	p 0.5439	p 0.0730	p 0.5439
	CI (-0.1662, 0.301)	CI (-0.4238, 0.02672)	CI (-0.1662, 0.301)
WBC	Spearman r 0.0497	Spearman r 0.1156	Spearman r 0.07168
	p 0.6731	p 0.3269	p 0.5439
	CI (-0.1807, 0.2753)	CI (-0.1161, 0.3353)	CI (-0.1662, 0.301)
PLT	Spearman r 0.0497	Spearman r 0.1156	Spearman r 0.07168
	p 0.6731	p 0.3269	p 0.5439
	CI (-0.1807, 0.2753)	CI (-0.1161, 0.3353)	CI (-0.1662, 0.301)
LYMPH	Spearman r -0.1456	Spearman r -0.1198	Spearman r -0.1198
	p 0.2157	p 0.3095	p 0.3095
	CI (-0.3681, 0.0926)	CI (-0.3451, 0.1186)	CI (-0.3451, 0.1186)
NEUTR	Spearman r 0.05989	Spearman r 0.1431	Spearman r -0.01169
	p 0.6122	p 0.2239	p 0.9213
	CI (-0.1709, 0.2845)	CI (-0.0883, 0.3598)	CI (-0.2396, 0.2174)

**Table 6:** Statistical correlations among different variables in epitheloid GBM patients. GBM - glioblastoma multiforme; NLR - neutrophil-to-lymphocyte ratio; PLR - platelet-to-lymphocyte ratio; MLR - monocyte-to-lymphocyte ratio; ELR - eosinophil-to-lymphocyte ratio; SII - systemic immune-inflammatory index; SIRI - systemic immune response index; WBC - absolute count of white blood cells; LYMPH - absolute count of lymphocytes; PLT - absolute count of platelets; NEUTR - absolute count of neutrophils

GBM epitheloid	Age	Dimension	Preoperative KPSI
NLR	Spearman r 0.1088	Spearman r 0.03865	Spearman r -0.07684
	p 0.6573	p 0.8752	p 0.7545
	CI (-0.3759, 0.5469)	CI (-0.4349, 0.4955)	CI (-0.5238, 0.4033)
MLR	Spearman r 0.4193	Spearman r 0.2855	Spearman r -0.2258
	p 0.5420	p 0.2361	p 0.3526
	CI (-0.3401, 0.575)	CI (-0.2079, 0.663)	CI (-0.6257, 0.2681)
PLR	Pearson r -0.04942	Pearson r 0.00898	Spearman r 0.1734
	p 0.8408	p 0.9709	p 0.4779
	CI (-0.4927, 0.4142)	CI (-0.4472, 0.4614)	CI (-0.3181, 0.5913)
ELR	Spearman r -0.08823	Spearman r -0.04053	Spearman r -0.01153
	p 0.7194	p 0.8691	p 0.9626
	CI -0.5321, 0.3927)	CI (-0.4969, 0.4334)	CI (-0.4747, 0.4566)
SII	Pearson r 0.1276	Pearson r 0.07358	Spearman r -0.02999
	p 0.6025	p 0.7647	p 0.903
	CI (-0.3468, 0.5501)	CI (-0.3939, 0.5108)	CI (-0.4889, 0.4419)
SIRI	Spearman r 0.2871	Spearman r 0.3118	Spearman r -0.2333
	p 0.2234	p 0.1937	p 0.3364
	CI (-0.2062, 0.664)	CI (-0.1801, 0.6789)	CI (-0.6305, 0.2607)
WBC	Pearson r 0.1895	Pearson r 0.1731	Spearman r -0.105
	p 0.4372	p 0.4786	p 0.6689
	CI (-0.2898, 0.5928)	CI (-0.3052, 0.5816)	CI (-0.5441, 0.3793)
PLT	Pearson r -0.1654	Pearson r 0.1252	Spearman r 0.02624
	p 0.4986	p 0.6094	p 0.9151
	CI (-0.5764, 0.3124)	CI (-0.3489, 0.5484)	CI (-0.4449, 0.486)
LYMPH	Spearman r 0.1111	Spearman r 0.08822	Spearman r -0.1035
	p 0.6507	p 0.7195	p 0.6733
	CI (-0.3614, 0.5381)	CI (-0.3813, 0.5215)	CI (-0.5327, 0.368)
NEUTR	Pearson r 0.1594	Pearson r 0.1404	Spearman r -0.06692
	p 0.5144	p 0.5664	p 0.7855
	CI (-0.3178, 0.5722)	CI (-0.3352, 0.559)	CI (-0.5058, 0.3994)

**Table 7:** Statistical correlations among different variables in NOS GBM patients. NOS - not otherwise specified; GBM - glioblastoma multiforme; NLR - neutrophil-to-lymphocyte ratio; PLR - platelet-to-lymphocyte ratio; MLR - monocyte-to-lymphocyte ratio; ELR - eosinophil-to-lymphocyte ratio; SII - systemic immune-inflammatory index; SIRI - systemic immune response index; WBC - absolute count of white blood cells; LYMPH - absolute count of lymphocytes; PLT - absolute count of platelets; NEUTR - absolute count of neutrophils

GBM NOS	Age	Dimension	Preoperative KPS
NLR	Spearman r 0.06412	Spearman r 0.3212	Spearman r 0.01334
	p 0.7021	p 0.0493	p 0.9366
	CI (-0.2701, 0.3845)	CI (-0.008221, 0.5877)	CI (-0.3166, 0.3404)
MLR	Spearman r 0.05787	Spearman r 0.1695	Spearman r 0.004822
	p 0.73	p 0.3089	p 0.9771
	CI (-0.2759, 0.3792)	CI (-0.1684, 0.4718)	CI (-0.3242, 0.3328)
PLR	Spearman r -0.02992	Pearson r 0.1773	Spearman r -0.02411
	p 0.8585	p 0.2869	p 0.8858
	CI (-0.355, 0.3016)	CI (-0.151, 0.4704)	CI (-0.3499, 0.3068)
ELR	Spearman r -0.1107	Spearman r -0.3018	Spearman r 0.2053
	p 0.5081	p 0.0655	p 0.2163
	CI (-0.4238, 0.226)	CI (-0.5735, 0.02963)	CI (-0.1321, 0.5001)
SII	Spearman r 0.009974	Spearman r 0.1383	Spearman r 0.01873
	p 0.9526	p 0.4077	p 0.9111
	CI (-0.3196, 0.3374)	CI (-0.1993, 0.4465)	CI (-0.3117, 0.3451)
SIRI	Spearman r 0.07782	Spearman r 0.1839	Spearman r 0.04889
	p 0.6424	p 0.2691	p 0.7707
	CI (-0.2573, 0.3962)	CI (-0.154, 0.4832)	CI (-0.2842, 0.3715)
WBC	Spearman r 0.04275	Spearman r 0.1198	Spearman r 0.1211
	p 0.7989	p 0.4738	p 0.4691
	CI (-0.2898, 0.3661)	CI (-0.2173, 0.4313)	CI (-0.2161, 0.4324)
PLT	Spearman r -0.1096	Pearson r -0.2945	Spearman r 0.01301
	p 0.5124	p 0.0727	p 0.9382
	CI (-0.4229, 0.2271)	CI (-0.5614, 0.02789)	CI (-0.3169, 0.3401)
LYMPH	Spearman r 0.0134	Spearman r -0.2754	Spearman r 0.02115
	p 0.936	p 0.0942	p 0.8997
	CI (-0.3075, 0.3317)	CI (-0.5469, 0.04856)	CI (-0.3006, 0.3385)
NEUTR	Spearman r 0.05952	Spearman r 0.1443	Spearman r 0.02673
	p 0.7226	p 0.3874	p 0.8734
	CI (-0.2652, 0.3721)	CI (-0.1839, 0.4435)	CI (-0.2955, 0.3435)

**Table 8:** Statistical correlations among different variables in IDHwt GBM patients. IDHwt - isocitrate dehydrogenase wild type; GBM - glioblastoma multiforme; NLR - neutrophil-to-lymphocyte ratio; PLR - platelet-to-lymphocyte ratio; MLR - monocyte-to-lymphocyte ratio; ELR - eosinophil-to-lymphocyte ratio; SII - systemic immune-inflammatory index; SIRI - systemic immune response index; WBC - absolute count of white blood cells; LYMPH - absolute count of lymphocytes; PLT - absolute count of platelets; NEUTR - absolute count of neutrophils

GBM IDHwt	Age	Dimension	Preoperative KPSI
NLR	Spearman r 0.5364	Spearman r -0.1644	Spearman r -0.2581
	p 0.0939	p 0.6337	p 0.4348
	CI (-0.1141, 0.8469)	CI (-0.7062, 0.4988)	CI (-0.7521, 0.4215)
MLR	Pearson r 0.01669	Pearson r -0.1747	Pearson r 0.08353
	p 0.9612	p 0.6074	p 0.8071
	CI (-0.5892, 0.6106)	CI (-0.7012, 0.4751)	CI (-0.5437, 0.6509)
PLR	Pearson r 0.38	Pearson r -0.1669	Pearson r -0.3641
	p 0.249	p 0.6237	p 0.271
	CI (-0.285, 0.798)	CI (-0.6971, 0.4813)	CI (-0.7912, 0.3018)
ELR	Spearman r -0.0694	Spearman r 0.1195	Spearman r -0.05472
	p 0.8385	p 0.7345	p 0.8812
	CI (-0.6545, 0.5677)	CI (-0.5324, 0.6825)	CI (-0.646, 0.5776)
SII	Pearson r 0.1338	Pearson r -0.2398	Pearson r -0.181
	p 0.6948	p 0.4776	p 0.5943
	CI (-0.5068, 0.6793)	CI (-0.7342, 0.4207)	CI (-0.7045, 0.47)
SIRI	Spearman r 0.3909	Spearman r -0.3881	Spearman r -0.1721
	p 0.2366	p 0.2366	p 0.6147
	CI (-0.292, 0.8098)	CI (-0.8087, 0.295)	CI (-0.7101, 0.4929)
WBC	Pearson r -0.08452	Pearson r -0.2627	Pearson r 0.06257
	p 0.8048	p 0.4351	p 0.8550
	CI (-0.6515, 0.543)	CI (-0.7452, 0.4004)	CI (-0.5584, 0.6386)
PLT	Spearman r -0.2273	Spearman r 0.1644	Spearman r -0.239
	p 0.5034	p 0.6337	p 0.4684
	CI (-0.7375, 0.4481)	CI (-0.4988, 0.7062)	CI (-0.7431, 0.4382)
LYMPH	Spearman r 0.05124	Spearman r -0.2708	Spearman r 0.004061
	p 0.8811	p 0.4206	p 0.9905
	CI (-0.566, 0.6317)	CI (-0.749, 0.3929)	CI (-0.5973, 0.6025)
NEUTR	Pearson r 0.03383	Pearson r -0.3663	Pearson r 0.00679
	p 0.9213	p 0.2679	p 0.9843
	CI (-0.5778, 0.6211)	CI (-0.7921, 0.2994)	CI (-0.5956, 0.6042)

Our results revealed a weak positive association between the NOS group's tumor size and NLR values (Spearman r 0.3212, p 0.0493). No other correlations proved statistically significant.

Using Kaplan-Meier survival curves and log-rank tests, we evaluated the influence of every serum biomarker on patients' overall survival. We did not find statistical significance when applying this statistical analysis to the entire GBM population. However, when assessing each molecular subtype individually, we did obtain a statistically significant influence of the total number of WBC on the overall survival rate in NOS GBMs, HR 0.4153, 95% CI 0.1825-0.9449, p 0.0362. No other significant differences have been found among the studied groups regarding survival.

## Discussion

GBMs are the most infamous primary malignancies affecting the adult brain. Accessible and accurate factors to predict disease evolution and, consequently, to permit an individualized approach to each patient are highly desirable yet difficult to identify. Immuno-oncology is an emerging domain that studies the interactions between tumors and the immune system, which can further be utilized in developing strategies for diagnosis, prognosis, and even treatment [11]. Serum biomarkers mirroring the immune/inflammatory response status in predicting GBM evolution could be a promising field [11]. Lately, numerous studies have questioned their reliability, but the results are conflicting. In our study, we primarily evaluated the role of various serum immune-inflammatory biomarkers in the survival of GBM patients: NLR, PLR, MLR, ELR, SII, SIRI, WBC, NEUTR, PLT, and LYMPH. Our results revealed that increased WBC at the moment of diagnosis could predict a lower overall survival.

NLR is a commonly cited biomarker and, in some contexts, is used to quantify the host response to cancer development. Neutrophils reflect the inflammatory reaction, whereas lymphocytes' behavior correlates with the immune response. A high NLR means a prevalence of the inflammatory response, which is more permissive to cancer progression, and, consequently, might predict a worse prognosis [5]. However, the available literature presents contradictory results. Subeikshanan et al. [12] compared NLR values between patients diagnosed with intra- and extra-axial brain tumors prior to receiving any type of treatment with healthy controls, and they discovered a more pronounced increase in NLR level, particularly in the GBM subpopulation.

Further studies confirmed that elevated preoperative NLR values are associated with more aggressive GBMs and shorter survival [2,13,14]. Lei et al. [2] suggested a more prolonged adjuvant chemotherapy cure in patients with increased preoperative NLR, as they are considered at high-risk for failure. Haksoyler et al. [15] concluded that patients with low pre-treatment NLR responded better to pharmacological intervention with bevacizumab and irinotecan regarding overall survival. Moreover, NLR could be utilized in grading glioma, as higher NLR values are associated with increasing WHO grade [8,14,16]. Gan et al. [17] concluded that high NLR carries a poor prognosis for elderly patients with high-grade gliomas. Although our study failed to confirm any influence of NLR on the overall survival of GBM patients, we did discover higher NLR levels in older patients in the IDHwt subgroup.

Furthermore, we found a weak positive association between tumor size and NLR levels. A previous study on a similar cohort demonstrated that the GBM dimension is an unfavorable prognostic marker for patients' survival [18]. This implies that NLR might carry a prognostic significance, although no statistically significant conclusion can be drawn to support it. Other studies confirm NLR's prognostic role in predicting GBM patients' survival [19,20]. Brenner et al. [5] found no significant correlation between NLR and overall survival, but age was one of the main factors predicting a poor outcome. Paradoxically, Lopes et al. [21] reported that a lower NLR is associated with lower progression-free survival, but they also demonstrated that a higher NLR relates to worse overall survival in patients undergoing the Stupp protocol. Even though NLR could be an affordable prognostic marker in individualizing the therapeutic approach in GBM patients, its utility is still debatable, as existing studies have inconsistent results. One of the disadvantages might be the lack of a recognized cut-off value since it largely varies among studies.

An absolute number of platelets and PLR have been utilized as markers of inflammation, although less extensively than NLR. Platelets have a salutary effect on tumor growth by enhancing the cancer cells' escape from the immune system. Furthermore, they release molecules, such as vascular endothelial growth factor (VEGF) and platelet-derived growth factor (PDGF), that stimulate cell proliferation and metastasis [19]. Consequently, Baran et al. [22] have suggested the importance of PLR in differentiating between GBM and brain metastasis, although less accurately than lymphocyte-to-monocyte ratio (LMR); they demonstrated a higher PLR and a lower LMR in brain metastasis. Other studies and our current study refuted any influence of PLR on overall survival [10,21]. Kaya et al. [13] reported a trend toward shorter survival in patients with higher PLR but did not prove it statistically. On the other hand, Wang et al. [14] statistically demonstrated a shorter survival in patients with increased PLR values, however, Cox regression did not sustain PLR as an independent prognostic factor for glioma patients. 

Monocytes might be considered valuable tools in predicting tumor grading in the future. More aggressive tumors contain large areas of necrosis that release inflammatory molecules and, consequently, trigger a more powerful immune response: not only do they increase peripheral neutrophils at the expense of circulating lymphocytes, but they also decrease peripheral monocytes by recruiting them within the tumor surroundings [11]. Zhang et al. [23]have demonstrated that monocytes have a prognostic role in gliomas by modulating the immune response and enhancing both tumor growth and invasion capacity. Existing studies yield conflicting results: while some [3] have proven that low MLR values are associated with longer overall survival in GBM patients, others [19]have contested any statistically significant association between MLR and progression-free survival or overall survival. As mentioned above, LMR might be used as a valuable biomarker to distinguish metastasis from GBMs [22] and to predict the grade of gliomas [16]. We tried to find if there is any influence of MLR on patients' survival in our group, yet nothing statistically significant has emerged.

Eosinophils are generally known as markers of anti-parasitic and allergic reactions. Additionally, they might be implicated in tumorigenesis, although their precise role is still debatable: in some solid tumors, namely lung and colon cancer, they inhibit tumor growth, while Hodgkin lymphoma eosinophil infiltration carries an unfavorable prognosis [4]. It seems that the risk of gliomas in patients with active asthma is lower than in those with inactive asthma, which might imply a protective role of eosinophils [24]. The precise mechanisms are still under investigation but are most probably related to the molecules stored within the eosinophils. For instance, major basic protein (MBP) and eosinophil cationic protein (ECP) have cytotoxic properties by damaging the cell membrane [4]. Eosinophil-derived neurotoxin (EDN) facilitates eosinophil infiltration in GBM tissue by binding to toll-like receptor-2 [4].

Furthermore, eosinophils also release cytokines that modulate the immune response: Th1-associated cytokines have anti-tumor features, whereas Th2-associated cytokines are associated with poor prognoses [4]. Huang et al. [4] proved lower absolute eosinophil count and ELR in higher-grade gliomas, GBM included. Madhugiri et al. [25] also concluded that the absolute eosinophil number is greater in GBM patients with better prognosis. Surprisingly, in our GBM cohort, patients with better survival tended to have lower ELR before any treatment was administered, but these results were not statistically proven. Further studies are necessary to elucidate the precise role of eosinophils and ELR In GBM development.

Total WBC has recognized prognostic value in cardiovascular and cerebrovascular disorders. It also proved useful in prostate cancer risk and prognosis [1]. Dubinski et al. [26] demonstrated that dexamethasone-induced leukocytosis is associated with a poorer prognosis in GBM patients. Their explanation for these results consisted of dexamethasone's influence on the behavior of the different leukocytes that already occupied the tumor surroundings. Consequently, they recommend reducing the dosage of the steroid treatment to the necessary minimum [26].

Nonetheless, studies focusing on leukocytosis influence on GBM patients' survival before receiving any treatment are scarce. Our results showed reduced overall survival in patients with increased WBC, and survival curve analysis revealed leukocytosis as an unfavorable prognosis biomarker in the NOS subpopulation. Maas et al. [20]found that an increased preoperative WBC led to decreased survival in GBM patients; nonetheless, multivariate analysis disproved the results. Similar results were described by Yang et al. [9].

SIRI and SII are derived biomarkers from the ratios above. Shi et el. [19] reported SII as the only serum biomarker to be an independent prognostic marker for progression-free and overall survival. SII broadly reflects the immunological status. A higher SII suggests a perturbance in the equilibrium between pro- and anti-tumorigenic factors favoring the first. Neutrophils impede the cytolytic activity of monocytes and release molecules that stimulate angiogenesis (e.g., VEGF) and tumor growth (e.g., neutrophil elastase). Elevated platelet count also favors tumor development via the mechanisms mentioned before. Lymphocytes, which would have displayed protective anti-tumor immunologic behavior, are in reduced amounts and, therefore, insufficient to fight against tumor growth [19]. Yang et al. [9] also reported SII as an independent prognostic biomarker for GBM overall survival, confirmed by both uni- and multivariate analysis.

On the contrary, Yilmuz et al. [27] performed a multivariate analysis that argued against SII as an independent prognostic factor for progression-free and overall survival in GBM patients. We found no statistical significance concerning the SII influence on GBM outcome. SIRI has also been questioned as a prognostic factor for GBM. Topkan et al. [28] stated that increased SIRI values are associated with shorter overall survival in newly diagnosed patients treated with Stupp protocol. Other publications also confirmed that high preoperative SIRI predicts a worse outcome in GBM patients [29,30]. Shi et al. [19]contradicted these findings. Although our study failed to demonstrate that SIRI is a useful prognostic factor, our results did show that patients with higher SIRI also had a larger tumor, which is understandable as elevated neutrophil and monocyte counts favor tumor growth [19,23]. Furthermore, increased SIRI values might be associated with a worse clinical status of the patients.

Study limitations

One of our study's main recognized limitations is the reduced number of patients due to single-center implementation. A regional or national study would have permitted a superior coverage of GBM pathology with a broader selection of patients and a much more even distribution within various subpopulations for further statistical assessment. Moreover, because of the retrospective nature of the study, precise information related to previous disorders or treatment administered prior to receiving GBM diagnosis and omitted to be recorded, which could have influenced the current results, is lacking. Furthermore, the determination of other inflammatory markers, such as C reactive protein (CRP) and erythrocyte sedimentation rate (ESR), would have allowed a broader perspective on the immune-inflammatory status of each patient. Another important drawback is the absence of worldwide established cut-off values for each biomarker, which would permit a reproducible statistical analysis among different centers.

## Conclusions

Finding accurate and affordable prognostic markers remains an important goal in glioblastoma management. Serum biomarkers would be the most accessible as complete blood counts are performed on all GBM patients before applying any surgical and/or pharmacological treatment. Nonetheless, their prognostic role is still debatable. Our study showed that an increased number of white blood cells prior to any therapeutic intervention could predict reduced overall survival. However, further investigations are warranted to establish whether the preoperative immune-inflammatory status of GBM patients could reliably predict their evolution.
